# An annotated checklist of Coccinellidae with four new records from Pakistan (Coleoptera, Coccinellidae)

**DOI:** 10.3897/zookeys.803.22543

**Published:** 2018-12-06

**Authors:** Muhammad Ali, Khalil Ahmed, Shaukat Ali, Ghulam Raza, Ishtiaq Hussain, Maisoor Ahmed Nafees, Syed Ishtiaq Anjum

**Affiliations:** 1 Department of Biological Sciences, University of Baltistan, Skardu, Gilgit-Baltistan, Pakistan; 2 Department of Biological Sciences, Karakoram International University, Gilgit, Gilgit-Baltistan, Pakistan; 3 Department of Environmental Science, Karakoram International University, Gilgit, Gilgit-Baltistan, Pakistan; 4 Department of Agriculture, District Kharmang, Gilgit-Baltistan, Pakistan; 5 Department of Zoology, Kohat University of Science and Technology, Kohat, Khyber Pakhtunkhwa, Pakistan

**Keywords:** aphid, Chilocorinae, Coccidulinae, Coccinellinae, mealybug, predatory, Coccinellinae, Scymninae

## Abstract

Some new ladybird (Coleoptera: Coccinellidae) records collected during the last four years across Sindh are reported. A first preliminary checklist of ladybirds from Sindh is presented, consisting of one subfamily, ten tribes, 21 genera, and 29 species including four new records, namely *Bulaealichatschovii* (Hummel), *Exochomuspubescens* Küster, Scymnus (Pullus) latemaculatus Motschulsky, Scymnus (Pullus) syriacus Marseul, and four varieties of the species *Cheilomenessexmaculatus* (Fabricius).

## Introduction

According to the most recent classification, the family Coccinellidae comprises two subfamilies: Microweiseinae Leng, 1920 and Coccinellinae Latreille, 1807 (sensu Slipinski 2007) based on phylogenetic results (Seago et al. 2011). These changes impact the status of various traditionally recognized tribes and subfamilies, as the onlysubfamilies now recognized are Microweiseinae and Coccinellinae (Canepari et al. 2016). Microweiseinae comprises three tribes: Carinodulini, Microweiseini, and Serangiini whereas Coccinellinae consists of only two tribes: Coccinellini and Chilocorini (Robertson et al. 2015)

Worldwide, nearly 6000 species spanning nearly 360 genera are known. Approximately 90 % of the species are predators of aphids, coccids, psyllids, aleyrodids, chrysomelid larvae, and mites, the remainder being herbivorous or mycetophagous (Inayatullah 1984, Majerus 1994, Obrycki and Kring 1998, Iperti and Bertand 2001, Vandenberg 2002, Hodek 2012).The Coccinellidae are an important group of beetles from both an economic standpoint in their use as biological control agents and in their diversity and adaptation to a number of differing habitats (Michels 1987).

From Pakistan, Ahmad and Ghani (1966, 1968, 1970, 1973), Inayatullah and Siddiqui (1978, 1979, 1980), and Ali et al. (2012) worked on different species of the family Coccinellidae; Iablokoff-Khnzorian (1986) described a new species *Adaliapuetzi* from Pakistan; Hashmi and Tashfeen (1992) studied the coccinellids housed in different institutions of Pakistan and reported 162 species, identifying the coccinellids deposited in the Natural History Museum, London, but with wrong synonymies. The present authors tried to trace this valuable collection of coccinellids in the present institutions in Karachi and other cities of Pakistan but found very few coccinellids. The authors also tried to correct the wrong synonymies and wrong identifications mentioned in the above-mentioned paper with the help of checklists and taxonomic papers available. Irshad (2001) listed 71 species of coccinellids in Pakistan; Rafi et al. (2005) gave a brief external morphology of predatory coccinellids of northern parts of Pakistan with special reference to their hosts, prey and localities, and listed 37 genera and 75 species belonging to different tribes of subfamilies Chilocorinae, Coccidulinae, Coccinellinae, Scymninae, and Sticholotidinae. All listed species are very common in Pakistan and represent a much less complete inventory than that of Hashmi and Tashfeen (1992). Otherwise, the description of genitalia was totally absent. Ali et al. (2012, 2013, 2014, 2015) conducted a systematic study from Sindh Province for the first time. They listed 29 coccinellids with four new records and four varieties of *Cheilomenessexmaculatus*.

According to Ghouri 1960, Kazmi 1980, Hashmi et al. (1983), Ali and Munir 1984, Ghani 1985, Inayatullah 1984, Mohyuddin and Mahmood 1993, Buriro 1996, Jan et al. 2003, Aslam et al. 2004, Abbas et al. 2007, Solangi et al. 2007, Massod et al. 2008, Rafiq et al. 2008, Arif et al. 2009, Mari and Lohar 2010, Iqbal et al. 2008, Iqbal et al. 2011, and Masood 2011, the following viz., *Schizaphisgraminum* (Rondani), *Sitobionavenae* (Fabricius), *Aphisgossypii* Glover, *Aphisfabae* Scopoli, *Aphisnerii* Boyer de Fonscolombe, *Aphiscraccivora* (Koch) *Rhopalosiphummaidis* (Fitch), *Therioaphistrifolii* (Monell), *Hysteroneurasetariae* (Thomas), *Lipaphiserysimi* (Kaltenbach), *Brevicorynebrassicae* (Linnaeus), *Myzuspersicae* (Sulzer), and *Hyadaphiscoriandri* (Das) (Homoptera: Aphididae); *Amritodusatkinsoni* (Lethierry)), *Amrascabiguttulabiguttula* (Ishida), *Empoascalybica* (Bergevin and Zanon) (Homoptera: Cicadellidae); *Bemisiatabaci* (Gennadius), *Aleurolobusbarodensis* (Maskell), *Dialeurodescitri* (Ashmead) and *Aleurocanthushusaini* Corbett (Homoptera: Aleyrodidae); *Brevipalpuslewisi* McGregor (Acarina: Tenuipalpidae), *Eutetranychusorientalis* (Klein), and *Tetranychusatlanticus* McG. (Acarina: Tetranychidae) are common pests of wheat, cotton, sugarcane, mango, mustard, vegetables, and fruits in Pakistan. Other works related with the taxonomy, morphology, diversity, distribution and ecology of different coccinellids include Rahman (1940), Ahmad (1969), Irshad (2001b), Khan et al. (2006), Rahatullah et al. (2010, 2011, 2012); Ali et al. (2012); Abbas et al. (2013), and Ashfaque et al. (2013). Ali (2012, 2013, 2014, 2015) was the first to report 29 coccinellid species from Sindh with a brief study on the taxonomy of the family Coccinellidae and their role in the field of biological control of important agricultural crop pests such as aphids, mealybugs, scale insects, jassids, and whiteflies.

The coccinellid fauna of Sindh, Pakistan is insufficiently known, and no checklist exists. The goal of this paper is to contribute to the knowledge of diversity and distribution of ladybirds in Sindh as well as to present the first preliminary checklist of the species recorded previously in the territory of Sindh.

## Materials and methods

Ladybird records presented in this paper were collected, identified, and confirmed during the last four years by the authors following the checklists, descriptions, and keys given by Chapin and Ahmad (1966), Pang and Gordon (1986), Poorani (2004), and Rafi et al. (2005), and with the help of the following website: NBAIR (2009). Ladybirds were also identified and confirmed by Dr. Claudio Canepari (Societa Entomologica Italiana), an authority on the family Coccinellidae. Specimens were collected during field trips conducted in different parts of Sindh Province, and in reality represent random findings instead of systematic collecting. Beetles were collected in standard ways, including manual collecting, net sweeping, and using light traps. The terminologies for various taxonomic structures including genitalia and procedures used by Inayatullah and Siddiqui (1978) and Gordon (1985) were generally followed. The taxonomic structures, especially male and female genitalia, were preserved after illustration in microvials with glycerine and pinned with specimens.

## Results

The coccinellids present in this checklist are classified on the basis of the new classification given by Seago et al. 2011, Robertson et al. 2015, and Canepari 2016. According to this classification all the coccinellids of the Sindh Province belong to the subfamily Coccinellinae only. It includes nine species of the tribe Coccinellini, one species of the Psylloborini, one species of the tribe Bulaeini, five species of the Chilocorini, one species of the Tribe Noviini Mulsant, one species from Tribe Hyperaspini, one species from the Tribe Stethorini, six species of Scymnini, one species of the Tribe Shirozuellini, and three species of the Tribe Sticholotidini. New records are *Bulaealichatschovii* (Hummel), *Exochomuspubescens* Küster, Scymnus (Pullus) latemaculatus Motschulsky, Scymnus (Pullus) syriacus Marseul with four varieties of *Cheilomenessexmaculatus* (Fabricius).

### Subfamily Coccinellinae Latreille, 1807

#### Tribe Coccinellini Latreille, 1807

##### *Coccinella* Linnaeus, 1758

###### Coccinella septempunctata

Taxon classificationAnimaliaColeopteraCoccinellidae

Linnaeus, 1758

Fig. 1

####### General distribution.

India, Nepal, Sri Lanka, Pakistan, Palaearctic. North America (Poorani 2002).

####### Distribution in Sindh.

Tandojam, Larkana, Mirpur Khas, Thatta, Karachi (Sarwar 2009, Mahmood et al. 2011, Ali 2013, Fazal Ellahi et al. 2017).

####### Host plants and prey species in Sindh.

*Brevicorynebrassicae* (L), *Lipaphiserysimi* (Kaltenbach), *Myzuspersicae* (Sulzer), *Aphisgossypii* (Glover), *Hyadaphiscoriandri* (Das), *Hysteroneurasetariae* (Thomas), *Schizaphisgraminum* (Rondani) (Aphididae: Homoptera); *Phenacoccussolenopsis* (Tinsley), *Ferrisiavirigata* (Ckll) (Pseudococcidae: Homoptera); *Amrascadevastans* (Dist), *Amrascabiguttulabiguttula* (Ishida) (Cicadellidae: Homoptera); *Bemisiatabaci* (Gennadius) (Aleyrodidae: Homoptera) on mustard, lucern, cabbage, cauliflower, potato, turnip, bottle gourd, eggplant, okra, wheat, cotton, sugarcane, and rose plants (Ali 2013).

**Figure 1.**
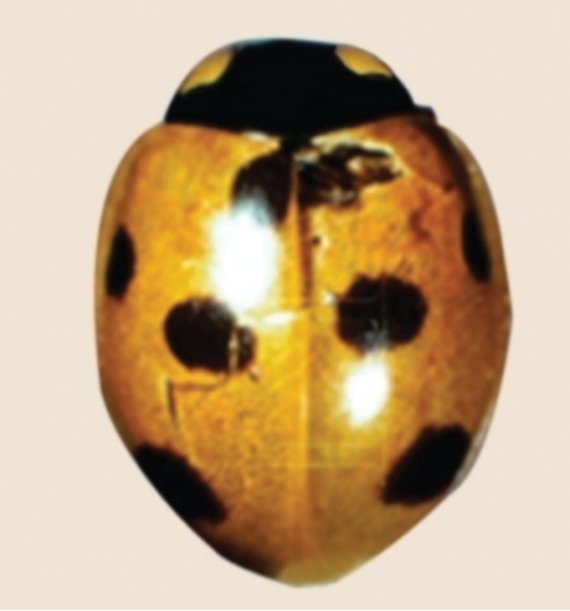
*Coccinellaseptempunctata* Linnaeus.

###### Coccinella undecimpunctata

Taxon classificationAnimaliaColeopteraCoccinellidae

Linnaeus, 1758

Fig. 2

####### General distribution.

India, Pakistan. Palaearctic (Poorani 2002).

####### Distribution in Sindh.

Karachi, Hyderabad, Tandojam, Mirpur Khas and Thatta (Sarwar 2009, Mahmood et al. 2011, Ali 2013, Fazal Ellahi et al. 2017).

####### Host plants and prey species in Sindh.

*Brevicorynebrassicae* (L.), *Lipaphiserysimi* (Kaltenbach), *Myzuspersicae* (Sulzer), *Aphisgossypii* (Glover), *Hyadaphiscoriandri* (Das), *Hysteroneura*setariae (Thomas), *Schizaphisgraminum* (Rondani) (Aphididae: Homoptera); *Phenacoccussolenopsis* (Tinsley), *Ferrisiavirigata* (Ckll) (Pseudococcidae: Homoptera); *Amrascadevastans* (Dist), *Amrascabiguttulabiguttula* (Ishida) (Cicadellidae: Homoptera); *Bemisiatabaci* (Gennadius) (Aleyrodidae: Homoptera) on mustard, lucern, cabbage, cauliflower, potato, turnip, bottle gourd, brinjal, okra, wheat, cotton, sugarcane, and rose plants (Ali 2013).

**Figure 2.**
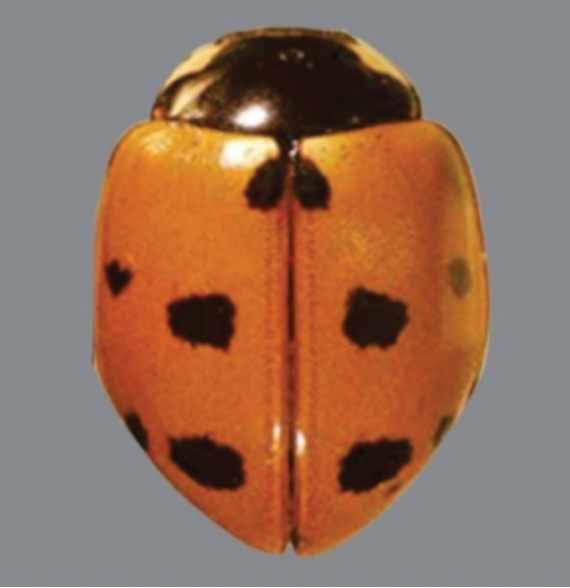
*Coccinellaundecimpunctata* Linnaeus.

###### Coccinella transversalis

Taxon classificationAnimaliaColeopteraCoccinellidae

Fabricius, 1781

Fig. 3

####### General distribution.

India, Nepal, Sri Lanka, Bangladesh, Indochina, Indonesia, Japan, Australia, New Zealand (Poorani 2002).

####### Distribution in Sindh.

Hyderabad, Larkana, Mirpur Khas, and Thatta (Ali 2013).

####### Host plants and prey species in Sindh.

*Brevicorynebrassicae* (L.), *Lipaphiserysimi* (Kaltenbach), *Myzuspersicae* (Sulzer), *Aphisgossypii* (Glover), *Hyadaphiscoriandri* (Das), *Hysteroneura*setariae (Thomas), *Schizaphisgraminum* (Rondani) (Aphididae: Homoptera); *Phenacoccussolenopsis* (Tinsley), *Ferrisiavirigata* (Ckll) (Pseudococcidae: Homoptera); *Amrascadevastans* (Dist), *Amrascabiguttulabiguttula* (Ishida) (Cicadellidae: Homoptera); *Bemisiatabaci* (Gennadius) (Aleyrodidae: Homoptera) on mustard, lucern, cabbage, cauliflower, potato, turnip, bottle gourd, brinjal, okra, wheat, cotton, sugarcane, and rose plants (Ali 2013).

**Figure 3.**
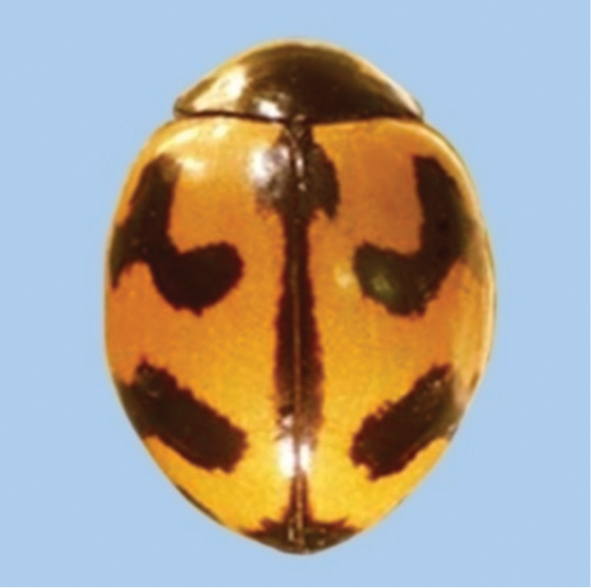
*Coccinellatransversalis* Fabricius.

##### *Cheilomenes* Dejean, 1836

###### Cheilomenes sexmaculata

Taxon classificationAnimaliaColeopteraCoccinellidae

(Fabricius, 1781)

Fig. 4

####### General distribution.

India, Bangladesh, Pakistan, Sri Lanka, Bhutan, Myanmar. Malaysia, Indonesia, Philippines, Vietnam, China, Japan, Australia (Poorani 2002).

####### Distribution in Sindh.

Hyderabad, Larkana, Mirpur Khas, and Thatta (Sarwar 2009, Mahmood et al. 2011, Ali 2013, Balouchi and Swati 2014, Fazal Ellahi et al. 2017).

####### Host plants and prey species in Sindh.

*Aphiscraccivora* Koch, *A.gossypii* Glover, *Brevicorynebrassicae* (L.), *Lipaphiserysimi* (Kaltenbach), *Myzuspersicae* (Sulzer), *Aphisgossypii* (Glover), *Hyadaphiscoriandri* (Das), *Hysteroneura*setariae (Thomas), *Schizaphisgraminum* (Rondani), *Ropalosiphummaidis* (Fitch), *Therioaphistrifolii* Monell (Aphididae: Homoptera); *Phenacoccussolenopsis* (Tinsley), *Ferrisiavirigata* (Ckll), *Centrococcusinsolitus* Green (Pseudococcidae: Homoptera), *Drosichamangiferae* (Green) (Margarodidae: Homoptera) *Aleurocanthushusaini* Corbett, *Aleurocanthuswoglumi* Ashby, *Aleurolobusbarodensis* Mask *Amrascadevastans* (Dist), *Amrascabiguttulabiguttula* (Ishida), *Amritodusatkinsoni* Leth, *Evacanthusrepexus* Dist (Cicadellidae: Homoptera); *Bemisiatabaci* (Gennadius) (Aleyrodidae: Homoptera), *Pyrillaperpusilla* Walk (Fulgoridae: Homoptera), *Quadraspidiotusperniciosus* Comst (Diaspididae: Homoptera), *Diaphorinacitri* Kuw (Psyllidae: Homoptera), *Tetranychusorientalis* Mog (Acarina: Tetranychidae) on mustard, lucern, cabbage, cauliflower, potato, turnip, bottle gourd, eggplant, okra, wheat, cotton, and rose plants (Ali 2013).

####### Comment.

Common. It is very difficult to compare this species with other taxa because of polymorphism. Six varieties of this species are reported from Pakistan.

**Figure 4.**
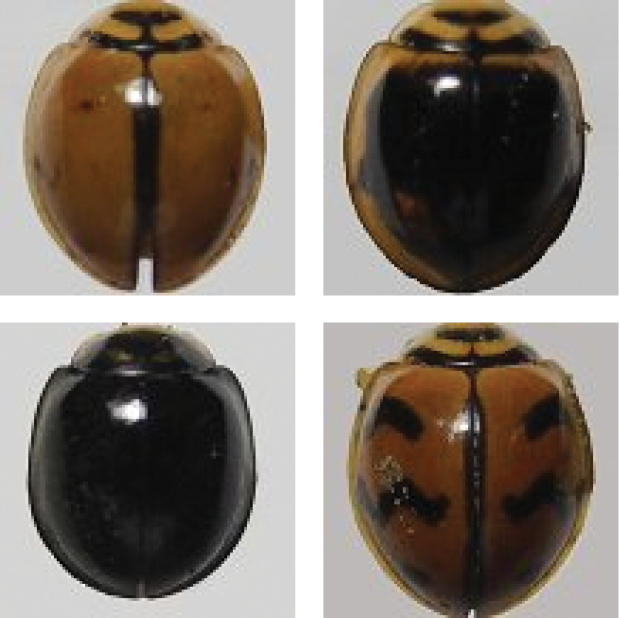
*Cheilomenessexmaculata* (Fabricius).

###### Hippodamia variegata

Taxon classificationAnimaliaColeopteraCoccinellidae

(Goeze, 1777)

Fig. 5

####### General distribution.

Nepal, Pakistan, Afghanistan, Tibet, Mongolia, China, northern and eastern Africa, Palaearctic (Poorani 2002).

####### Distribution in Sindh.

Hyderabad, Karachi, and Thatta (Lohar et al. 2012, Ali 2013).

####### Host plants and prey species in Sindh.

*Brevicorynebrassicae* (L.), *Lipaphiserysimi* (Kaltenbach), *Myzuspersicae* (Sulzer), *Aphisgossypii* (Glover), *Hyadaphiscoriandri* (Das), *Hysteroneura*setariae (Thomas), *Schizaphisgraminum* (Rondani) (Aphididae: Homoptera); *Phenacoccussolenopsis* (Tinsley), *Ferrisiavirigata* (Ckll) (Pseudococcidae: Homoptera); *Amrascadevastans* (Dist), *Amrascabiguttulabiguttula* (Ishida) (Cicadellidae: Homoptera); *Bemisiatabaci* (Gennadius) (Aleyrodidae: Homoptera) on mustard, lucern, cabbage, cauliflower, potato, turnip, bottle gourd, brinjal, okra, wheat, cotton, and rose plants (Ali 2013).

**Figure 5.**
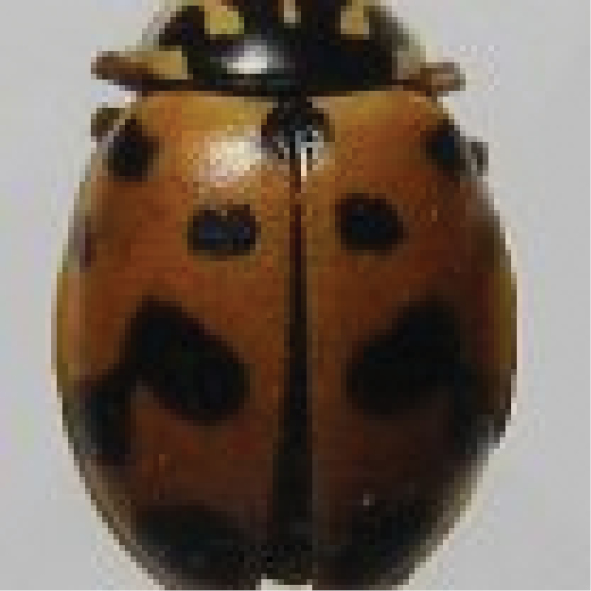
*Hippodamiavariegata* (Goeze).

###### Micraspis allardi

Taxon classificationAnimaliaColeopteraCoccinellidae

(Mulsant, 1866)

Fig. 6

####### General distribution.

India, Nepal, Pakistan, Myanmar, Indonesia (Poorani 2002).

####### Distribution in Sindh.

Hyderabad, Mirpur Khas, Thatta and Karachi (Ali 2013).

####### Host plants and prey species in Sindh.

*Amritodusatkinsoni* Teth (Cicadellidae: Homoptera) *Quadraspidiotusperniciosus* Comst (Diaspididae: Homoptera), *Pyrillaperpusilla* Walk (Fulgoridae: Homoptera) (Ali 2013).

**Figure 6.**
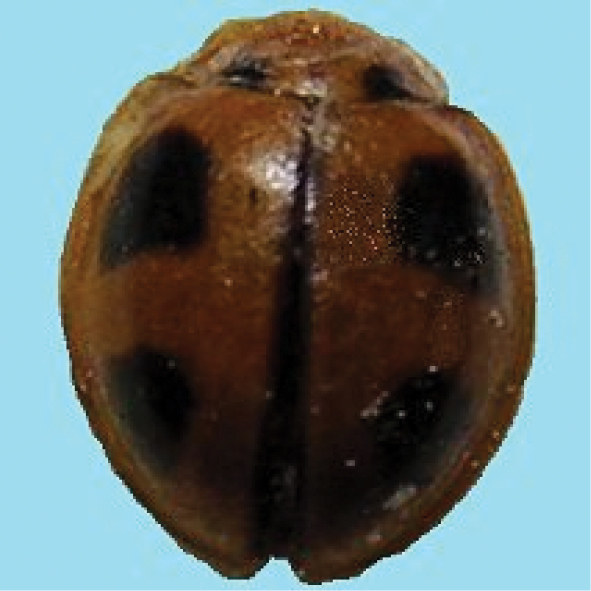
*Micraspisallardi* (Mulsant).

###### Oenopia sauzeti

Taxon classificationAnimaliaColeopteraCoccinellidae

Mulsant, 1866

Fig. 7

####### General distribution.

India, Bhutan, Pakistan, Nepal, Myanmar, Thailand, China (Poorani 2002).

####### Distribution in Sindh.

Hyderabad, Mirpur Khas, Thatta, and Karachi (Ali 2013).

####### Host plants and Prey species in Sindh.

*Aphiscraccivora* Koch, *A.gossypii* Glover, *Brevicorynebrassicae* (L.), *Lipaphiserysimi* (Kaltenbach), *Myzuspersicae* (Sulzer), *Aphisgossypii* (Glover), *Hyadaphiscoriandri* (Das), *Schizaphisgraminum* (Rondani), *Ropalosiphummaidis* (Fitch) (Aphididae: Homoptera), *Aleurolobusbarodensis* Mask *Amrascadevastans* (Dist), *Amrascabiguttulabiguttula* (Ishida), *Evacanthusrepexus* Dist (Cicadellidae: Homoptera), *Tetranychus* sp. (Acarina: Tetranychidae) on wheat, mustard, and cabbage (Ali 2013).

**Figure 7.**
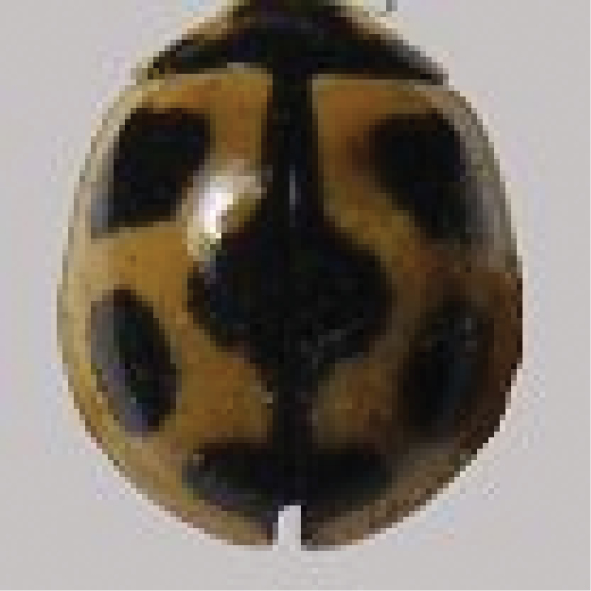
*Oenopiamimica* Weise.

###### Propylea quatuordecimpunctata

Taxon classificationAnimaliaColeopteraCoccinellidae

(Linnaeus, 1758)

Fig. 8

####### General distribution.

India, Pakistan, Bangladesh, Japan, China, Europe, North America (Poorani 2002).

####### Distribution in Sindh.

Hyderabad and Karachi (Ali 2013).

####### Host plants and prey species in Sindh.

*Aphiscraccivora* Koch, *A.gossypii* Glover, *Brevicorynebrassicae* (L.), *Lipaphiserysimi* (Kaltenbach), *Myzuspersicae* (Sulzer), *Aphisgossypii* (Glover), *Hyadaphiscoriandri* (Das) (Aphididae: Homoptera), *Aleurolobusbarodensis* Mask *Amrascadevastans* (Dist), *Amrascabiguttulabiguttula* (Ishida), *Evacanthusrepexus* Dist (Cicadellidae: Homoptera) (Ali 2013).

**Figure 8.**
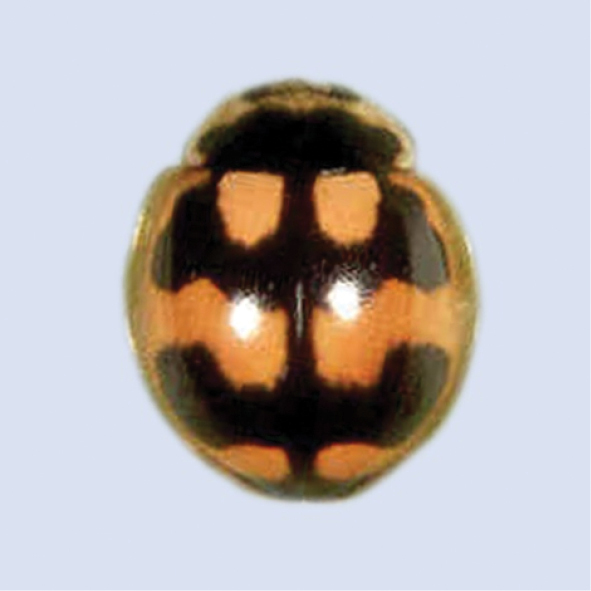
*Propyleaquatuordecimpunctata* (Linnaeus).

###### Harmonia dimidiata

Taxon classificationAnimaliaColeopteraCoccinellidae

(Fabricius, 1781)

Fig. 9

####### General distribution.

India, Pakistan, Nepal, Bhutan, China, Japan, Taiwan, introduced into North America (Poorani 2002).

####### Distribution in Sindh.

Hyderabad and Karachi (Ali 2013).

####### Host plants and prey species in Sindh.

*Aphiscraccivora* Koch, *A.gossypii* Glover, *Brevicorynebrassicae* (L), *Lipaphiserysimi* (Kaltenbach), *Myzuspersicae* (Sulzer), *Hyadaphiscoriandri* (Das), *Hysteroneurasetariae* (Thomas), *Ropalosiphummaidis* (Fitch), *Therioaphistrifolii* Monell, *Macrosiphumgranarium* (Kirby), *Schizaphisgraminum* (Rondani) (Aphididae: Homoptera), *Amritodusatkinsoni* Leth, *Idioscopusnagpurensis* Pruthi (Cicadellidae: Homoptera); *Bemisiatabaci* (Gennadius) (Aleyrodidae: Homoptera), *Tetranychusatlanticus* Mog (Acarina: Tetranychidae), *Adelges* spp. (Adelgidae: Homoptera) on mustard, lucern, cabbage, cauliflower, potato, turnip, bottle gourd, eggplant, okra, wheat, cotton, and rose plants (Ali 2013).

**Figure 9.**
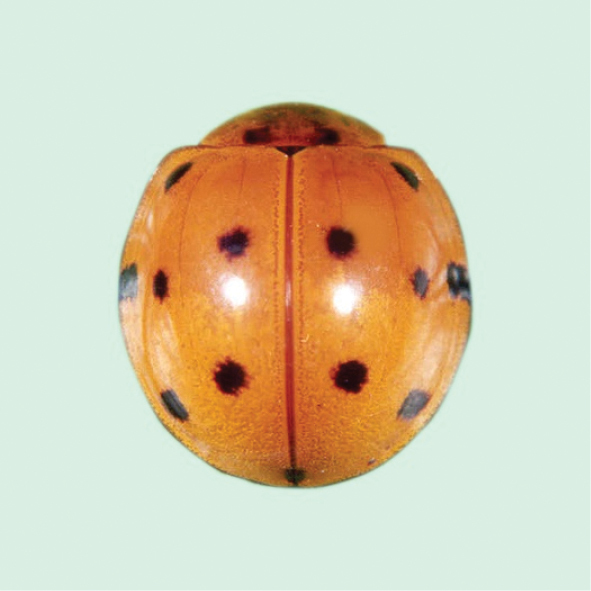
*Harmoniadimidiata* (Fabricius).

#### Tribe Bulaeini Savoiskaja, 1969

##### Bulaea lichatschovii

Taxon classificationAnimaliaColeopteraCoccinellidae

(Hummel, 1827)

Fig. 10

###### General distribution.

Pakistan, India, Central and West Asia, Afghanistan, Mediterranean region. North and Central Africa (Poorani 2002, Ali 2013).

###### Distribution in Sindh.

Hyderabad and Karachi (Ali 2013).

###### Host plants and prey species in Sindh.

*Aphiscraccivora* Koch, *A.gossypii* Glover, *Myzuspersicae* (Sulzer), *Diaphorinacitri* Kuw (Psyllidae: Homoptera) on wheat and mustard.

###### Comments.

Newly recorded from Pakistan.

**Figure 10.**
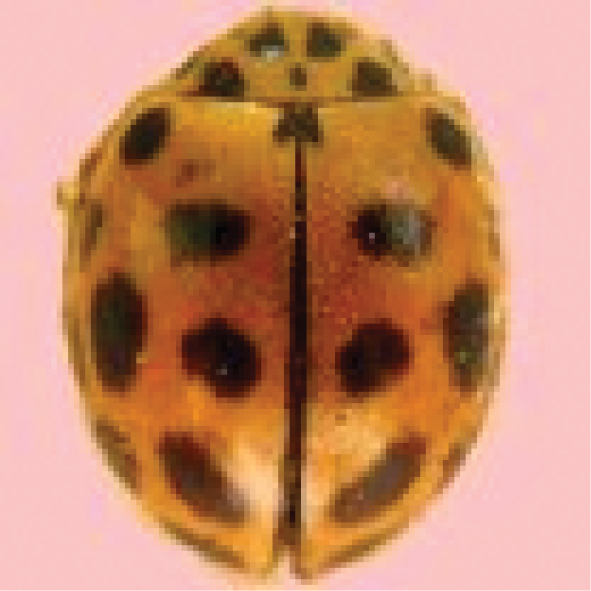
*Bulaealichatschovii* (Hummel).

#### Tribe Psylloborini Casey, 1899

##### Psyllobora bisoctonotata

Taxon classificationAnimaliaColeopteraCoccinellidae

(Mulsant, 1850)

Fig. 11

###### General distribution.

India and Pakistan (Poorani 2002).

###### Distribution in Sindh.

Hyderabad and Karachi (Ali 2013).

###### Prey in Sindh.

All the members of this genus are mycophagous (Ali 2013).

**Figure 11.**
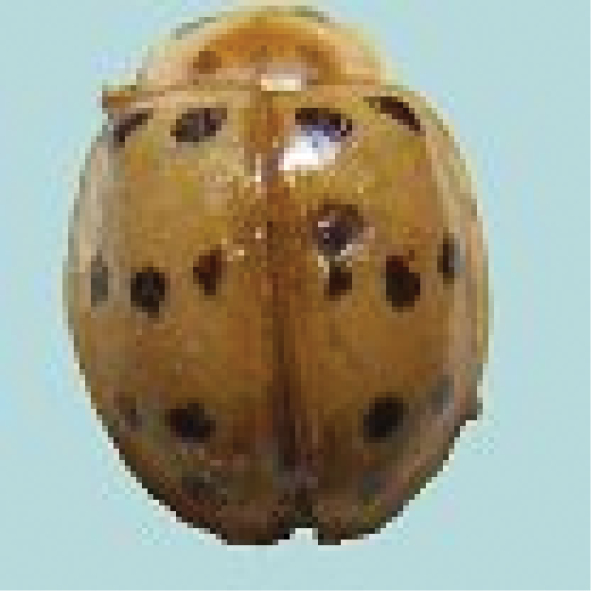
*Psylloborabisoctonotata* (Mulsant).

#### Tribe Chilocorini Costa, 1849

##### *Chilocorus* Leach, 1815b

###### Chilocorus nigrita

Taxon classificationAnimaliaColeopteraCoccinellidae

(Fabricius, 1798)

Fig. 12

####### General distribution.

Agalega, American Samoa, Burma, Brazil, Ghana, Guam, Hawaii, India, Indonesia, Kenya, Madagascar, Malaysia, Marshall Islands, New Caledonia, Nepal, Oman, Pakistan, Reunion Island, Seychelles, Solomon Islands, South Africa, Swaziland, Society Islands, Tanzania, Togo, Turkey and Zimbabwe (Nandwani and Joseph 2003, NBAII 2011, Omkar and Pervez 2003, Poorani 2002, Thomas and Blanchard 2014).

####### Distribution in Sindh.

Tandojam, Hyderabad and Karachi (Ali 2013).

####### Host plants and prey species in Sindh.

*Aonidiellaauranti* (Mask), *A.citrina* (Coq), *A.orientalis* Newst, *Aspidiotusdestructor* Sign, *Hemiberiesialatanias* (Sign), *Leucaspisconiferarum* Hall & Williams, *Parlatoria* spp, *Pinnaspisstrachani* (Cooley), *Quadraspidiotusperniciosus* Comst, *Tecaspis* spp. (Diaspididae: Homoptera) (Ali 2013).

**Figure 12.**
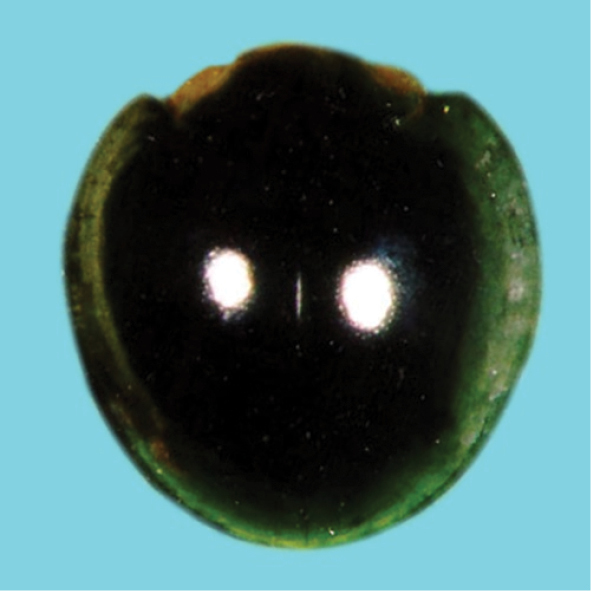
*Chilocorusnigrita* (Fabricius).

###### Exochomus (Parexochomus) nigripennis

Taxon classificationAnimaliaColeopteraCoccinellidae

Erichson, 1843

Fig. 13

####### General distribution.

northwestern India, Pakistan, Palaearctic, Africa (Poorani 2002).

####### Distribution in Sindh.

Tandojam, Mirpur Khas, Hyderabad, and Karachi (Ali, 2013).

####### Host plants and prey species in Sindh.

*Aphisfabae* Theobald, *Rhopalosiphummaidis* Fitch (Aphididae: Homoptera), *Parlatoria* spp. (Diaspididae: Homoptera), *Ferrisiavirigata* (Ckll) (Pseudococcidae: Homoptera). It was recorded on trees and wild plants (Ali 2013).

**Figure 13.**
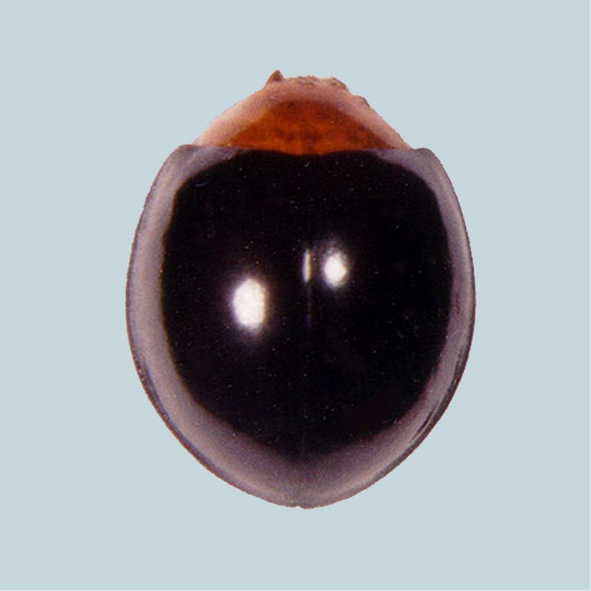
*Exochomusnigripennis* (Erichson).

###### Exochomus pubescens

Taxon classificationAnimaliaColeopteraCoccinellidae

Küster, 1848

Fig. 14

####### General distribution.

Pakistan, India, Spain, North Africa, Greece, Egypt, Syria, Palestine (Poorani 2002).

####### Distribution in Sindh.

Karachi (Ali 2013).

####### Host plants and prey species in Sindh.

*Parlatoria* spp. (Diaspididae: Homoptera). It was found on oak (Ali 2013).

####### Comment.

Newly recorded from Pakistan.

**Figure 14.**
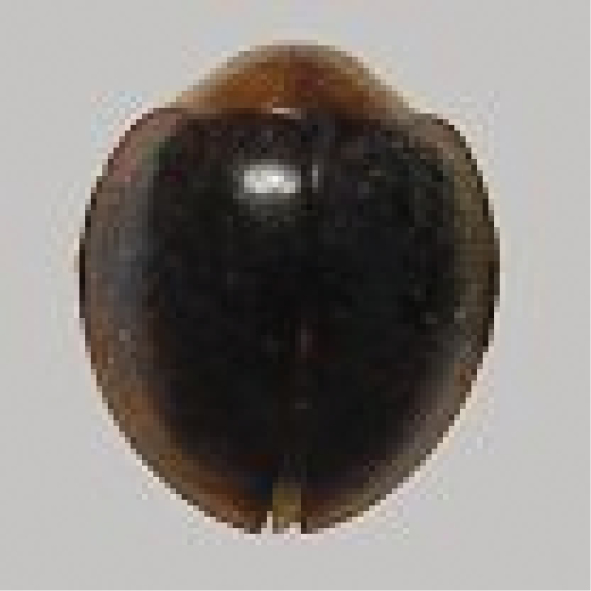
*Exochomuspubescens* Küster.

###### Priscibrumus uropygialis

Taxon classificationAnimaliaColeopteraCoccinellidae

(Mulsant, 1853)

Fig. 15

####### General distribution.

India, Bhutan, Pakistan, Nepal (Poorani 2002).

####### Distribution in Sindh.

Tandojam and Hyderabad (Ali 2013).

####### Host plants and prey species in Sindh.

*Parlatoria* spp., *Pinnaspisstrachani* (Cooley), *Quadraspidiotusperniciosus* Comst, *Tecaspis* spp. (Diaspididae: Homoptera) on wild trees, and shrubs (Ali 2103).

**Figure 15.**
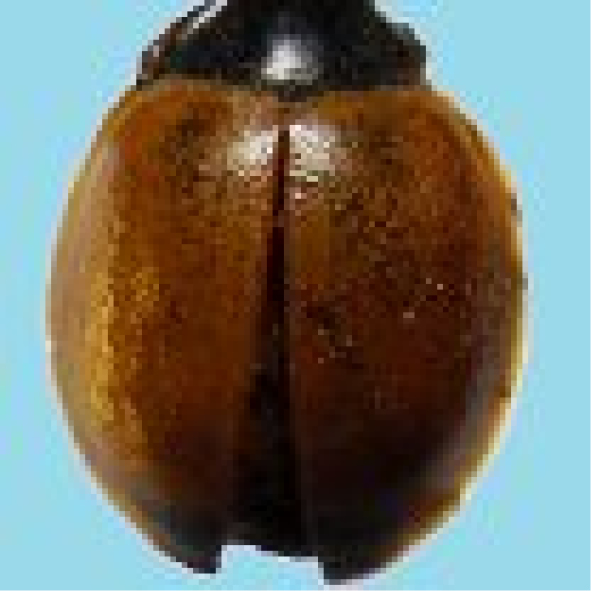
*Priscibrumusuropygialis* (Mulsant).

###### Brumoides suturalis

Taxon classificationAnimaliaColeopteraCoccinellidae

(Fabricius, 1798)

Fig. 16

####### General distribution.

India, Pakistan, Bangladesh, Sri Lanka, Bhutan, Nepal (Poorani 2002).

####### Distribution in Sindh.

Tandojam, Mirpur Khas, Hyderabad, and Karachi (Ali 2013).

####### Host plants and prey species in Sindh.

*Aphiscraccivora* Koch, *A.gossypii* Glover, *Brevicorynebrassicae* (L.), *Lipaphiserysimi* (Kaltenbach), *Myzuspersicae* (Sulzer), *Hyadaphiscoriandri* (Das), *Hysteroneurasetariae* (Thomas), *Ropalosiphummaidis* (Fitch), *Therioaphistrifolii* Monell, *Macrosiphumgranarium* (Kby), *Schizaphisgraminum* (Rondani) (Aphididae: Homoptera); *Phenacoccussolenopsis* (Tinsley), *Ferrisiavirigata* (Ckll) (Pseudococcidae: Homoptera), *Drosichamangiferae* (Green) (Margarodidae: Homoptera), *Amrascadevastans* (Dist), *Amrascabiguttulabiguttula* (Ishida) (Cicadellidae: Homoptera); *Bemisiatabaci* (Gennadius) (Aleyrodidae: Homoptera), *Tetranychusatlanticus* Mog (Acarina: Tetranychidae), *Adelgesjoshii* S.O & S (Adelgidae: Homoptera), *Aonidiellaauranti* (Mask), *A.citrina* (Coq), *A.orientalis* Newst, *Aspidiotusdestructor* Sign, *Hemiberiesialatanias* (Sign), *Leucaspisconiferarum* Hall & Williams, *Parlatoria* spp, *Pinnaspisstrachani* (Cooley), *Quadraspidiotusperniciosus* Comst, *Tecaspis* spp. (Diaspididae: Homoptera) on mustard, lucern, cabbage, cauliflower, potato, turnip, bottle gourd, eggplant, okra, wheat, cotton, and rose plants (Ali 2013).

**Figure 16.**
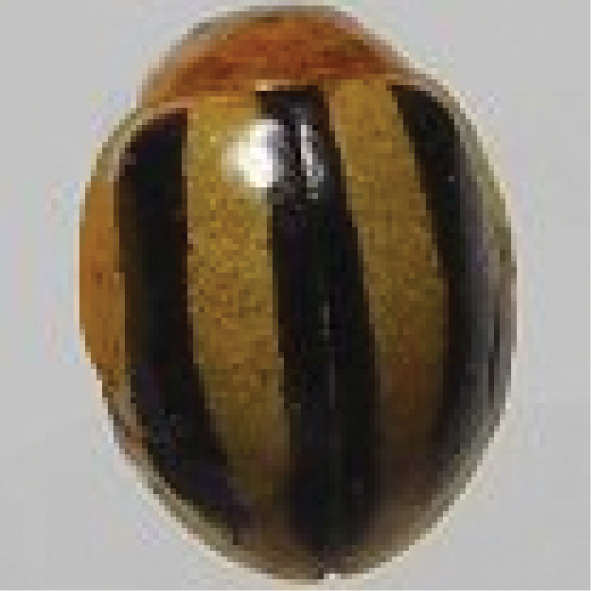
*Brumoidessuturalis* (Fabricius).

#### Tribe Noviini Mulsant, 1850, Genus *Rodolia* Mulsant, 1850

##### Rodolia ruficollis

Taxon classificationAnimaliaColeopteraCoccinellidae

Mulsant, 1850

Fig. 17

###### General distribution.

India, Pakistan, Thailand (Poorani 2002).

###### Distribution in Sindh.

Karachi and Mirpur Khas (Ali 2013).

###### Host plants and prey species in Sindh.

*Iceryaaegyptiaca* (Dougl) (Margarodidae: Homoptera). It was found on cotton and roses (Ali 2013).

**Figure 17.**
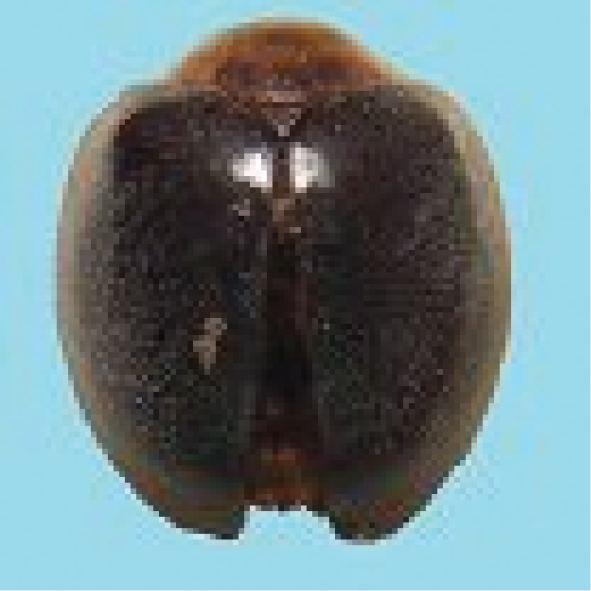
*Rodoliaruficollis* Mulsant.

#### Tribe Hyperaspini Costa, 1849, Genus *Hyperaspis* Chevrolat, 1836

##### Hyperaspis maindroni

Taxon classificationAnimaliaColeopteraCoccinellidae

Sicard, 1929

Fig. 18

###### General distribution.

Pakistan and India (Poorani 2002).

###### Distribution in Sindh.

Tandojam, Mirpur Khas, and Karachi (Ali 2013).

###### Host plants and prey species in Sindh.

*Centrococcusinsolitus* (Green), *Naiacoccus* sp, *Phenacoccussolenopsis* (Tinsley), *Ferrisiavirigata* (Ckll) (Pseudococcidae: Homoptera), *Drosichamangiferae* (Green) (Margarodidae: Homoptera). It was found on cotton, okra, and trees (Ali 2013).

**Figure 18.**
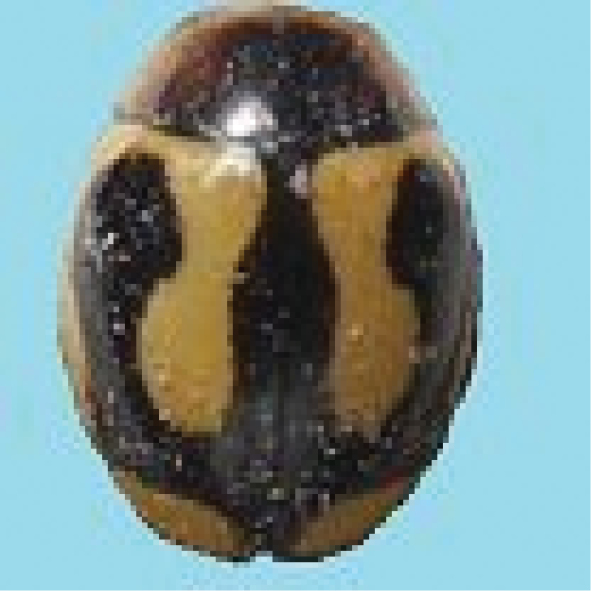
*Hyperaspismaindroni* Sicard.

#### Tribe Stethorini Dobzhansky, 1924, Genus *Stethorus* Weise, 1885b

##### Stethorus gilvifrons

Taxon classificationAnimaliaColeopteraCoccinellidae

(Mulsant, 1850)

Fig. 19

###### General distribution.

India, Pakistan, Italy, Cyprus (Poorani 2002).

###### Distribution in Sindh.

Tandojam, Hyderabad, Mirpur Khas and Karachi (Ali 2013).

###### Host plants and prey species in Sindh.

*Brevipalpus* sp. (Tenuipalpidae: Acarina), *Eutetranychuscernus* (B&P), *E.orientalis* (Klein), *Tetranychusatlanticus* Mog (Acarina: Tetranychidae). It was collected from eggplant, okra, and some wild plants (Ali 2013).

**Figure 19.**
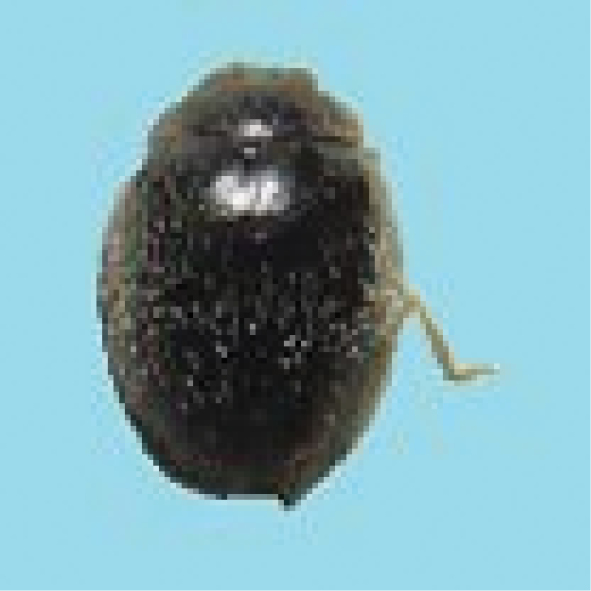
*Stethorusgilvifrons* (Mulsant).

#### Tribe Scymnini Mulsant, 1846, Genus *Scymnus*, Mulsant, 1850

##### Scymnus (Scymnus) nubilus

Taxon classificationAnimaliaColeopteraCoccinellidae

Mulsant, 1850

Fig. 20

###### General distribution.

Pakistan, India, Bangladesh, Sri Lanka, Nepal, Myanmar, China, Asia Minor (Poorani 2002).

###### Distribution in Sindh.

Tandojam, Mirpur Khas, Hyderabad, and Karachi (Ali 2013).

###### Host plants and prey species in Sindh.

*Aphiscraccivora* Koch, *A.gossypii* Glover, *Brevicorynebrassicae* (L.), *Lipaphiserysimi* (Kaltenbach), *Myzuspersicae* (Sulzer), *Aphisgossypii* (Glover), *Hyadaphiscoriandri* (Das), *Hysteroneura*setariae (Thomas), *Ropalosiphummaidis* (Fitch), *Therioaphistrifolii* Monell, *Macrosiphumgranarium* (Kby), *Schizaphisgraminum* (Rondani) (Aphididae: Homoptera); *Phenacoccussolenopsis* (Tinsley), *Ferrisiavirigata* (Ckll) (Pseudococcidae: Homoptera), *Drosichamangiferae* (Green) (Margarodidae: Homoptera), *Amrascadevastans* (Dist), *Amrascabiguttulabiguttula* (Ishida) (Cicadellidae: Homoptera); *Bemisiatabaci* (Gennadius) (Aleyrodidae: Homoptera), *Tetranychusatlanticus* Mog (Acarina: Tetranychidae), Adelgesjoshii S.O & S (Adelgidae: Homoptera), *Aonidiellaauranti* (Mask), *A.citrina* (Coq), *A.orientalis* Newst, *Aspidiotusdestructor* Sign, *Hemiberiesialatanias* (Sign), *Leucaspisconiferarum* Hall & Williams, *Parlatoria* spp, *Pinnaspisstrachani* (Cooley), *Quadraspidiotusperniciosus* Comst, *Tecaspis* spp. (Diaspididae: Homoptera) on mustard, lucern, cabbage, cauliflower, potato, turnip, bottle gourd, eggplant, okra, wheat, cotton and rose plants (Ali 2013).

**Figure 20.**
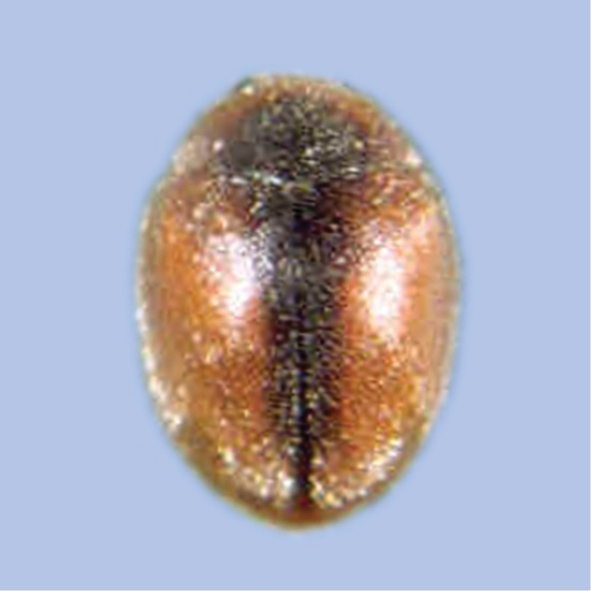
Scymnus (Scymnus) nubilus Mulsant.

##### Scymnus (Pullus) latemaculatus

Taxon classificationAnimaliaColeopteraCoccinellidae

Motschulsky, 1858

Fig. 21

###### General distribution.

Pakistan, India, Bangladesh, Sri Lanka, Thailand, Taiwan. (Poorani 2002; Ali 2013).

###### Distribution in Sindh.

Tandojam, Hyderabad, and Karachi (Ali 2013).

###### Host plants and prey species in Sindh.

*Aphiscraccivora* Koch, *A.gossypii* Glover, *Brevicorynebrassicae* (L.), *Lipaphiserysimi* (Kaltenbach), *Myzuspersicae* (Sulzer), *Aphisgossypii* (Glover), *Hyadaphiscoriandri* (Das), *Hysteroneura*setariae (Thomas), *Ropalosiphummaidis* (Fitch), *Therioaphistrifolii* Monell, *Macrosiphumgranarium* (Kby), *Schizaphisgraminum* (Rondani) (Aphididae: Homoptera); *Phenacoccussolenopsis* (Tinsley), *Ferrisiavirigata* (Ckll) (Pseudococcidae: Homoptera), *Drosichamangiferae* (Green) (Margarodidae: Homoptera), *Amrascadevastans* (Dist), *Amrascabiguttulabiguttula* (Ishida) (Cicadellidae: Homoptera); *Bemisiatabaci* (Gennadius) (Aleyrodidae: Homoptera), *Tetranychusatlanticus* Mog (Acarina: Tetranychidae) on mustard, lucern, cabbage, cauliflower, potato, turnip, bottle gourd, eggplant , okra, wheat, cotton, and rose plants (Ali 2013).

###### Comment.

Newly recorded from Pakistan.

**Figure 21.**
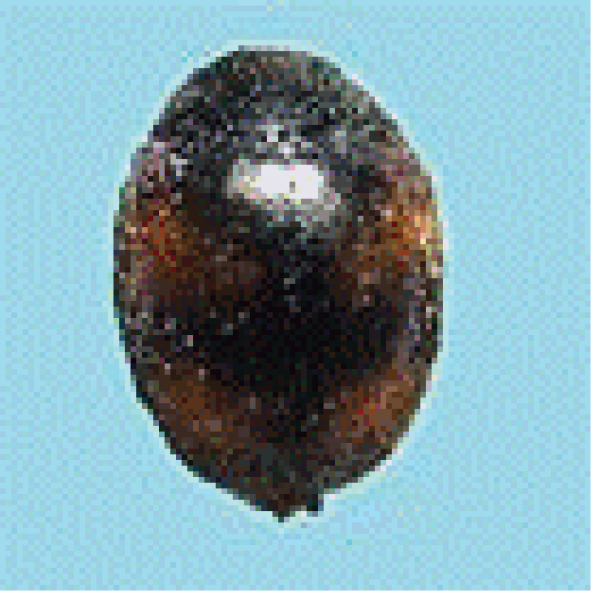
Scymnus (Pullus) latemaculatus Motschulsky.

##### Scymnus (Pullus) coccivora

Taxon classificationAnimaliaColeopteraCoccinellidae

Ayyar, 1925

Fig. 22

###### General distribution.

India, Pakistan, Bangladesh, Sri Lanka, Malaysia (Poorani 2002).

###### Distribution in Sindh.

Tandojam, Hyderabad, and Karachi (Ali 2013).

###### Host plants and prey species in Sindh.

*Aphiscraccivora* Koch, *A.gossypii* Glover, *Brevicorynebrassicae* (L.), *Lipaphiserysimi* (Kaltenbach), *Myzuspersicae* (Sulzer), *Aphisgossypii* (Glover), *Hyadaphiscoriandri* (Das), *Hysteroneura*setariae (Thomas), *Ropalosiphummaidis* (Fitch), *Therioaphistrifolii* Monell, *Macrosiphumgranarium* (Kby), *Schizaphisgraminum* (Rondani) (Aphididae: Homoptera); *Phenacoccussolenopsis* (Tinsley), *Ferrisiavirigata* (Ckll) (Pseudococcidae: Homoptera), *Drosichamangiferae* (Green) (Margarodidae: Homoptera), *Tetranychusatlanticus* Mog (Acarina: Tetranychidae) on mustard, lucern, cabbage, cauliflower, potato, turnip, bottle gourd, eggplant, okra, wheat, cotton, and rose plants (Ali 2013).

**Figure 22.**
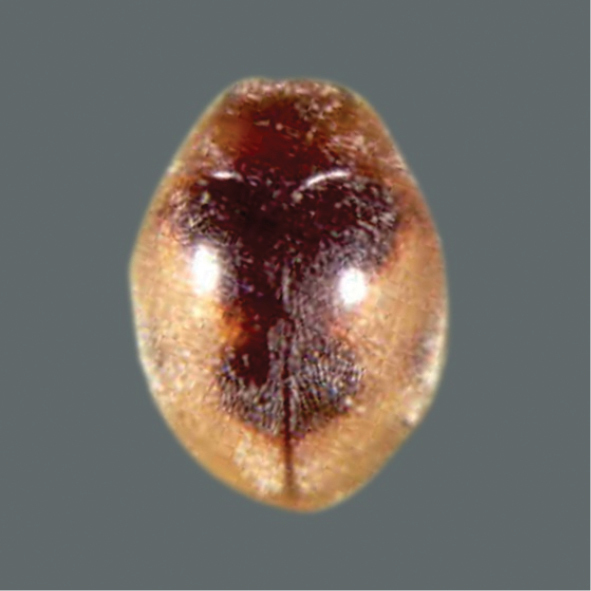
Scymnus (Pullus) coccivora Ayyar.

##### Scymnus (Pullus) castaneus

Taxon classificationAnimaliaColeopteraCoccinellidae

Sicard, 1929

Fig. 23

###### General distribution.

Pakistan, India, Bangladesh (Poorani 2002).

###### Distribution in Sindh.

Tandojam, Hyderabad and Karachi (Ali 2013).

###### Host plants and prey species in Sindh.

*Aphiscraccivora* Koch, *A.gossypii* Glover, *Brevicorynebrassicae* (L.), *Lipaphiserysimi* (Kaltenbach), *Myzuspersicae* (Sulzer), *Aphisgossypii* (Glover), *Hyadaphiscoriandri* (Das), *Hysteroneura*setariae (Thomas), *Ropalosiphummaidis* (Fitch), *Therioaphistrifolii* Monell, *Macrosiphumgranarium* (Kby), *Schizaphisgraminum* (Rondani) (Aphididae: Homoptera); *Phenacoccussolenopsis* (Tinsley), *Ferrisiavirigata* (Ckll) (Pseudococcidae: Homoptera), *Drosichamangiferae* (Green). It was found on eggplant, okra, cotton (Ali 2013).

###### Comment.

Newly recorded from Pakistan.

**Figure 23.**
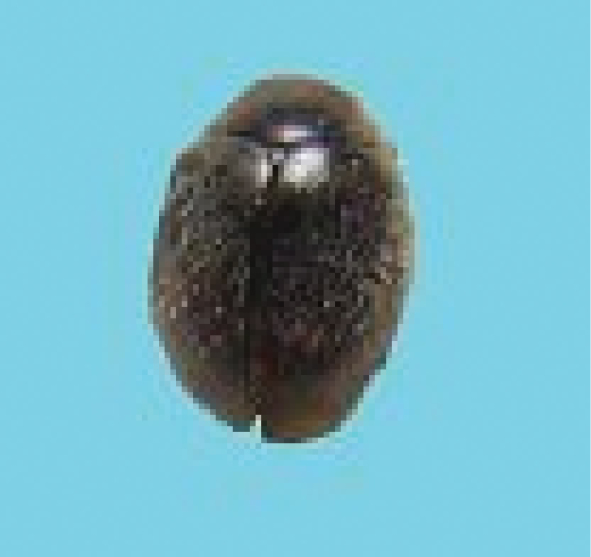
Scymnus (Pullus) castaneus Sicard.

##### Scymnus (Pullus) syriacus

Taxon classificationAnimaliaColeopteraCoccinellidae

(Marseul, 1868)

Fig. 24

###### General distribution.

Iran, Afghanistan, Pakistan (Ali 2013).

###### Distribution in Sindh.

Hyderabad and Karachi (Ali 2013).

###### Host plants and prey species in Sindh.

*Aphiscraccivora* Koch, *A.gossypii* Glover, *Brevicorynebrassicae* (L.), *Lipaphiserysimi* (Kaltenbach), *Myzuspersicae* (Sulzer), *Aphisgossypii* (Glover), *Hyadaphiscoriandri* (Das), *Hysteroneura*setariae (Thomas), *Ropalosiphummaidis* (Fitch), *Therioaphistrifolii* Monell, *Macrosiphumgranarium* (Kby), *Schizaphisgraminum* (Rondani) (Aphididae: Homoptera) (Ali 2013).

###### Comment.

Newly recorded from Pakistan.

**Figure 24.**
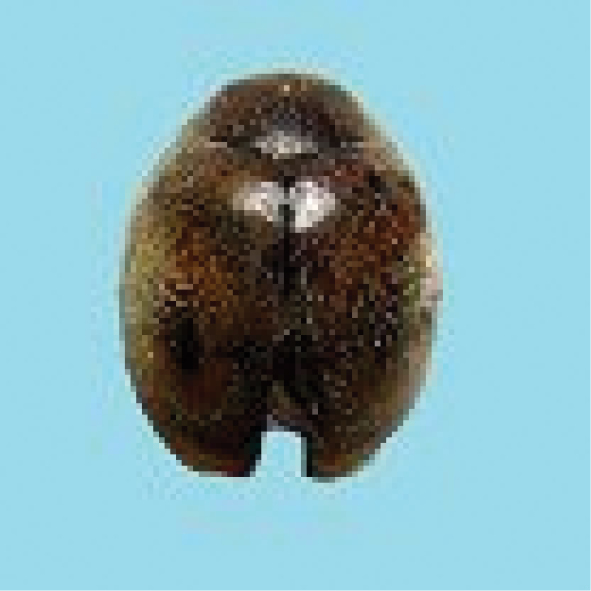
Scymnus (Pullus) syriacus (Marseul).

##### Nephus regularis

Taxon classificationAnimaliaColeopteraCoccinellidae

(Sicard, 1929)

Fig. 25

###### General distribution.

India, Pakistan, China (Poorani 2002).

###### Distribution in Sindh.

Tandojam, Mirpur Khas, Hyderabad and Karachi (Ali 2013).

###### Prey and host plant.

*Aphiscraccivora* Koch, *A.gossypii* Glover, *Aphisgossypii* (Glover), *Hyadaphiscoriandri* (Das), *Therioaphistrifolii* Monell (Aphididae: Homoptera); *Phenacoccussolenopsis* (Tinsley), *Ferrisiavirigata* (Ckll) (Pseudococcidae: Homoptera), *Drosichamangiferae* (Green) (Margarodidae: Homoptera) on on eggplant, okra and cotton (Ali 2013).

**Figure 25.**
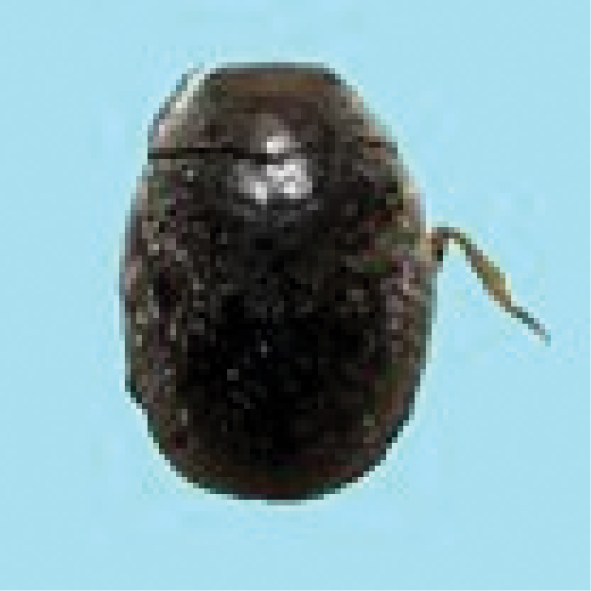
*Nephusregularis* (Sicard).

#### Tribe Shirozuellini Sasaji, 1967, Genus Ghanius Ahmad, 1973

##### Ghanius karachiensis

Taxon classificationAnimaliaColeopteraCoccinellidae

Ahmad, 1973

Fig. 26

###### General distribution.

Pakistan (Poorani 2002).

###### Distribution in Sindh.

Karachi (Ali 2013).

###### Host plants and prey species in Sindh.

*Aonidiellaauranti* (Mask), *A.citrina* (Coq), *A.orientalis* Newst, *Hemiberiesialatanias* (Sign), *Leucaspisconiferarum* Hall & Williams, *Parlatoria* spp. *Pinnaspisstrachani* (Cooley), *Quadraspidiotusperniciosus* Comst, *Tecaspis* spp. (Diaspididae: Homoptera) (Ali 2013).

**Figure 26.**
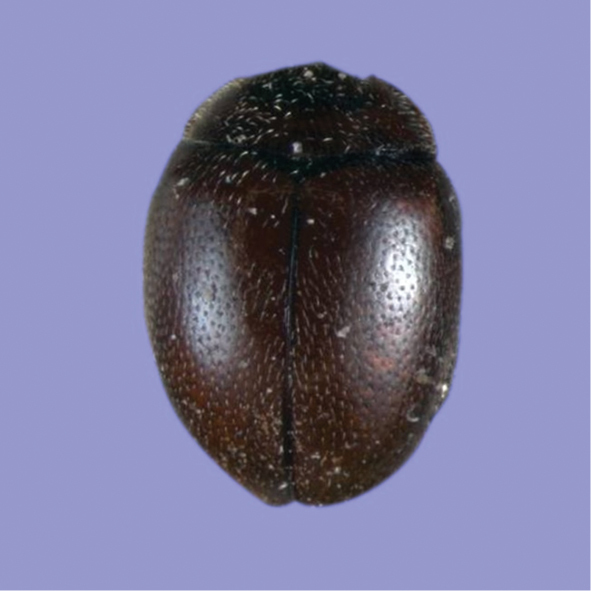
*Ghaniuskarachiensis* Ahmad.

#### Tribe Sticholotidini Weise, 1901

##### Pharoscymnus flexibilis

Taxon classificationAnimaliaColeopteraCoccinellidae

(Mulsant), 1853

Fig. 27

###### General distribution.

India, Pakistan, Brazil, and United States (Florida) (Poorani 2002, Thomas and Blanchard 2013).

###### Distribution in Sindh.

Tandojam, Mirpur Khas, Hyderabad, and Karachi (Ali 2013).

###### Host plants and prey species in Sindh.

*Aspidiotusdestructor* Sign, *Hemiberiesialatanias* (Sign), *Leucaspisconiferarum* Hall & Williams, *Parlatoria* spp, *Pinnaspisstrachani* (Cooley), *Quadraspidiotusperniciosus* Comst, *Tecaspis* spp. (Diaspididae: Homoptera), *Coccushesperidium* L, *Siassetianigra* (Nietn) (Coccidae: Homoptera) on wheat and mustard (Ali 2013).

**Figure 27.**
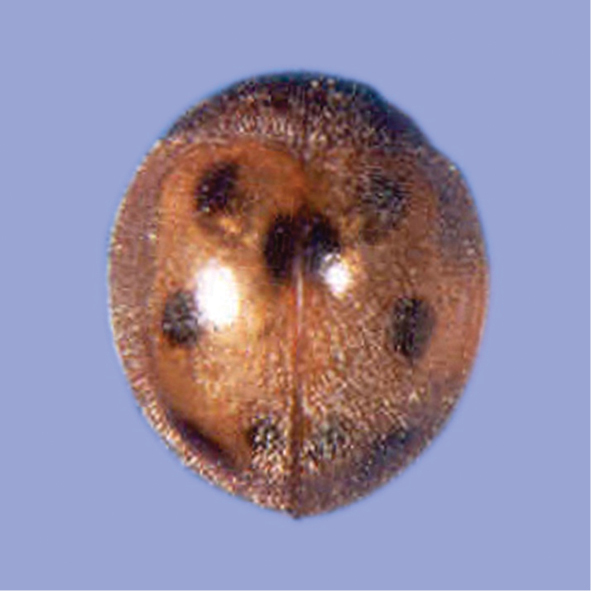
*Pharoscymnusflexibilis* (Mulsant).

##### Pharoscymnus simmondsi

Taxon classificationAnimaliaColeopteraCoccinellidae

Ahmad, 1970

Fig. 28

###### General distribution.

Pakistan, Thailand (Poorani 2002).

###### Distribution in Sindh.

Karachi (Ali 2013).

###### Host plants and prey species in Sindh.

*Parlatoria* spp., *Pinnaspisstrachani* (Cooley), *Quadraspidiotusperniciosus* Comst, *Tecaspis* spp. (Diaspididae: Homoptera), *Coccushesperidium* L, *Siassetianigra* (Nietn) (Coccidae: Homoptera) on wheat and mustard (Ali 2013).

**Figure 28.**
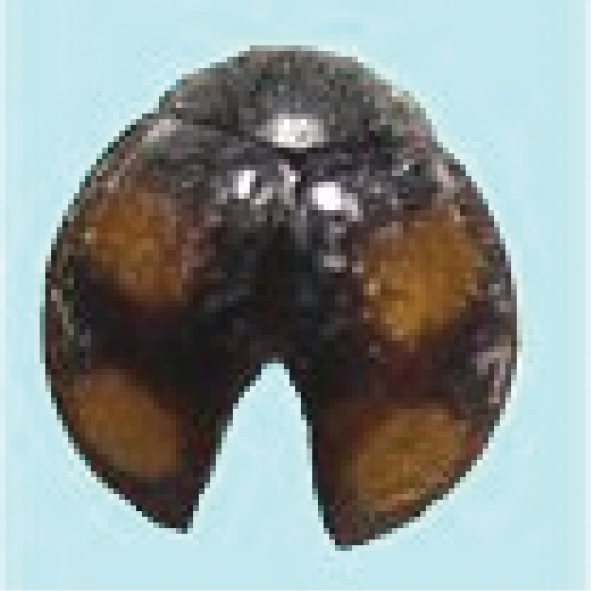
*Pharoscymnussimmondsi* Ahmad.

##### Pharoscymnus horni

Taxon classificationAnimaliaColeopteraCoccinellidae

(Weise), 1900

Fig. 29

###### General distribution.

India and Pakistan (Poorani 2002).

###### Distribution in Sindh.

Karachi (Ali 2013).

###### Host plants and prey species in Sindh.

*Parlatoria* spp. *Pinnaspisstrachani* (Cooley), *Quadraspidiotusperniciosus* Comst, *Tecaspis* spp. (Diaspididae: Homoptera), *Coccushesperidium* L, *Siassetianigra* (Nietn) (Coccidae: Homoptera) on mustard and wheat (Ali 2013).

**Figure 29.**
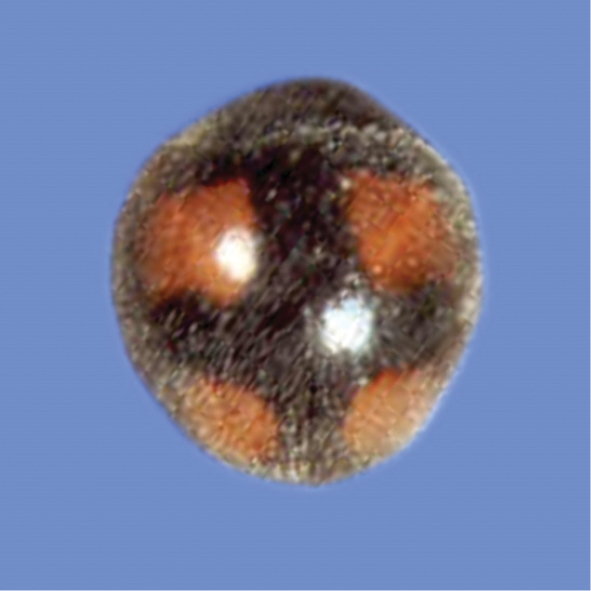
*Pharoscymnushorni* (Weise).

## Discussion

Unfortunately, all the specimens were lost during the shifting of Vitoria Museum to National Museum at Karachi. From Pakistan very little taxonomic work has focussed especially on this important family of the order Coleoptera. Irshad (2001) listed 71 species of coccinellids from northern parts of Pakistan. Rafi et al. (2005) listed 37 genera and 75 species and described the only external morphology of predatory coccinellids mostly collected from northern parts of Pakistan with special reference with their hosts, prey, and localities.

Sindh Province has a rich insect fauna which have diversified into important cities like Karachi, Tandojam, Hyderabad, Larkana, Sukhur, and Mirpur Khas. Coccinellids fauna is still incompletely recorded from Sindh region and has been neglected in the past. All the research findings on coccinellids except Ali (2013) were documentary not taxonomic. No proper collections, identification procedures,or techniques have been used in Sindh to explore the hidden records of insects, including the coccinellid fauna. Ali (2013) worked more comprehensively on the systematics and distribution of ladybirds of Sindh Province with reference to their role in biological control programmes. He tried to highlight the importance of systematic study to make easy their identification as predators of mealybugs, aphids, jassids, whiteflies, and scale insects. This research work may be useful for the entomologists including research students of particularly the Sindh region, but also of Pakistan and other Oriental regions. The geographical distribution and synonyms used in this study for all systematically treated specimens were cited from the findings of Hashmi and Tashfeen (1992).

The present investigation continues the research carried by Ali (2013), and gives a preliminary checklist of ladybirds from Sindh consisting of only one subfamily, ten tribes, 21 genera, and 29 species including four new records: *Bulaealichatschovii* (Hummel), *Exochomuspubescens* Küster, Scymnus (Pullus) latemaculatus Motschulsky, Scymnus (Pullus) syriacus Marseul and four varieties of *Menochilussexmaculata* (Fabricius). All these coccinellids from Pakistan are now placed into the subfamily Coccinellinae and the subfamily Microweiseinae according to the recent classification studies. The coccinellid specimens were deposited in the Natural History Museum, Department of Zoology, University of Karachi, Karachi, Pakistan.

## Supplementary Material

XML Treatment for Coccinella septempunctata

XML Treatment for Coccinella undecimpunctata

XML Treatment for Coccinella transversalis

XML Treatment for Cheilomenes sexmaculata

XML Treatment for Hippodamia variegata

XML Treatment for Micraspis allardi

XML Treatment for Oenopia sauzeti

XML Treatment for Propylea quatuordecimpunctata

XML Treatment for Harmonia dimidiata

XML Treatment for Bulaea lichatschovii

XML Treatment for Psyllobora bisoctonotata

XML Treatment for Chilocorus nigrita

XML Treatment for Exochomus (Parexochomus) nigripennis

XML Treatment for Exochomus pubescens

XML Treatment for Priscibrumus uropygialis

XML Treatment for Brumoides suturalis

XML Treatment for Rodolia ruficollis

XML Treatment for Hyperaspis maindroni

XML Treatment for Stethorus gilvifrons

XML Treatment for Scymnus (Scymnus) nubilus

XML Treatment for Scymnus (Pullus) latemaculatus

XML Treatment for Scymnus (Pullus) coccivora

XML Treatment for Scymnus (Pullus) castaneus

XML Treatment for Scymnus (Pullus) syriacus

XML Treatment for Nephus regularis

XML Treatment for Ghanius karachiensis

XML Treatment for Pharoscymnus flexibilis

XML Treatment for Pharoscymnus simmondsi

XML Treatment for Pharoscymnus horni
